# NK Cells, Monocytes and Macrophages in HIV-1 Control: Impact of Innate Immune Responses

**DOI:** 10.3389/fimmu.2022.883728

**Published:** 2022-05-27

**Authors:** Leonore Mensching, Angelique Hoelzemer

**Affiliations:** ^1^ Research Department Virus Immunology, Leibniz Institute of Virology (LIV), Hamburg, Germany; ^2^ I. Department of Internal Medicine, Division of Infectious Diseases, University Medical Center Hamburg-Eppendorf, Hamburg, Germany; ^3^ German Center for Infection Research (DZIF), Site Hamburg-Lübeck-Borstel-Riems, Hamburg, Germany

**Keywords:** HIV-1, elite control, NK cells, monocytes, macrophages, innate immunity, immune crosstalk

## Abstract

Rapid and synchronized responses of innate immune cells are an integral part of managing viral spread in acute virus infections. In human immunodeficiency virus type 1 (HIV-1) infection, increased immune control has been associated with the expression of certain natural killer (NK) cell receptors. Further, immune activation of monocytes/macrophages and the presence of specific cytokines was linked to low levels of HIV-1 replication. In addition to the intrinsic antiviral capabilities of NK cells and monocytes/macrophages, interaction between these cell types has been shown to substantially enhance NK cell function in the context of viral infections. This review discusses the involvement of NK cells and monocytes/macrophages in the effective control of HIV-1 and highlights aspects of innate immune crosstalk in viral infections that may be of relevance to HIV-1 infection.

## Introduction

The discovery of spontaneous HIV-1 control without antiretroviral therapy (ART) in people living with HIV-1 has initiated decades of research into the biological determinants of this observation, which - to date - have not been fully defined. Around 0.5% of all HIV-1 positive individuals belong to this group called elite controllers (EC). They are able to limit HIV-1 RNA viral load to less than 50 viral copies per mL in peripheral blood, maintain consistently high CD4+ T cell counts, and do not progress to develop acquired immunodeficiency syndrome (AIDS) (1).

Despite the heterogeneity of EC, their study has uncovered protective immune phenotypes and responses linked to better viral control. It is generally accepted that host factors have a stronger impact on HIV-1 control than viral factors (1, 2). Among these host factors are the presence of certain human leukocyte antigen (HLA) class I variants (3) and NK cell receptors (4), the ability to mount strong HIV-1 specific CD8+ T cell responses (5), and a limited proviral reservoir (6) in addition to several restriction factors (7).

A critical aspect that determines later HIV-1 control in some individuals is an effective early immune response towards the virus, restricting infection that may be mimicked by early ART intervention (8).

Innate sensing of HIV-1 triggers an inflammatory cascade involving macrophages, monocytes, and dendritic cells that activate T cells and other innate effectors, such as NK cells (9). The importance of NK cell and monocyte activation during hyperacute phases of HIV-1 infection was outlined recently by Kazer et al. in 2020 (10), emphasizing a potential association of an early and consistent innate immune response with HIV-1 control. Although antiviral functions of these innate immune cells are well described in a general context, their specific actions during acute HIV-1 infection and subsequently their impact on HIV-1 control demand further investigation. Here, associations between immune responses and effective control of HIV-1 from NK cells, monocytes, and macrophages are discussed. Interactions and crosstalk of these innate players are often overlooked, but may offer new perspectives on the establishment of long-term viral control.

## NK Cells, Monocytes and Macrophages in HIV-1 Control

### NK Cells as Innate Effectors in HIV-1 Control

In viral infections, NK cells are among the first immune cells to respond. There is increasing evidence for an important role of NK cells in HIV-1 disease progression and control (11–13). NK cells are innate cytotoxic effector lymphocytes that express a variety of activating and inhibitory receptors enabling them to detect virus-infected or transformed cells. Killer immunoglobulin-like receptors (KIR) are an NK cell receptor family that binds to HLA class I molecules. Downregulation of HLA class I by HIV-1 on infected cells increases their susceptibility towards NK cell-mediated killing (14–16). The detection of infected cells triggers the release of cytotoxic granules and initiates the production of cytokines. Furthermore, NK cells are able to mediate antibody dependent cell-mediated cytotoxicity (ADCC) *via* FcγRIII (CD16).

HIV-1 disease progression has been linked to certain protective NK cell receptors binding HLA class I (for a detailed review see Ref. (17)). Most prominent is the genetic association between heterozygosity of an activating KIR *KIR3DS1* with *HLA-B* alleles harboring a HLA-Bw4-I80 motif and slower HIV-1 disease progression (18). Although a first report examining the expression of protective KIRs and ligand combinations (KIR3DL1, KIR3DS1, HLA-Bw4-I80) in EC did not detect increased frequencies (19), a subsequent study by Tomescu et al. (20) found that NK cells from EC with a high expressing inhibitory KIR3DL1 together with HLA-Bw4-I80 ligands showed increased degranulation and cytokine production towards target cells. Additionally, NK cells from healthy donors expressing high levels of KIR3DL1 and HLA-Bw4-I80 are highly reactive against HIV-1 infected autologous CD4+ T cells *in vitro* (21). In a cohort of untreated HIV-1 controllers and non-controllers possessing the protective HLA-B*57 allele, Martin et al. (4) found a KIR3DL1 variant with an amino acid substitution at position 47 (I47V) that significantly enhanced the protective effect of HLA-B*57:01 but not that of HLA-B*57:03 further highlighting the importance of the KIR3DL1-HLA-B interaction in HIV-1 control.

The description of the nonclassical HLA class I molecule HLA-F as a high affinity ligand for KIR3DS1 (22) shed new light onto the association of *KIR3DS1* with slower HIV-1 disease progression. Infected CD4+ T cells upregulate HLA-F mRNA and are effectively killed by KIR3DS1+ NK cell clones (22) proposing an additional detection mechanism for HIV-1 infected cells by NK cells.

Further protective NK cell responses involve the activating receptors NKp44 and NKG2D. NKp44 has been implicated in the loss of CD4+ T cells and increased viral loads (23). In EC, NK cells do not upregulate NKp44 expression after stimulation by interleukin (IL)-2 compared to non-controllers, while still developing an activated and mature phenotype with expression of NKG2D and intact cytolytic function (24). The selective tuning of the NKp44 pathway might be an additional characteristic of NK cells that favors the maintenance of high CD4+ T cell counts, a hallmark of elite control.

It was shown that NK cells can kill HIV-1 infected CD4+ T cells *via* NKG2D (25). The action of the HIV-1 protein Vpr leads to upregulation of NKG2D ligands. To counteract this and escape NK cell-mediated killing, the HIV-1 protein Nef downregulates NKG2D ligands (26). Interestingly, in a single EC cohort study, HIV-1 Nef variants isolated from EC were found to be ineffective at downregulating NKG2D ligands on CD4+ T cells (27). With higher NKG2D surface expression of NK cells in EC (28, 29) and the fact that NKG2D also serves as a co-receptor for NK cell-mediated ADCC in HIV-1 (30), HIV-1 infected cells in EC may be particularly sensitive to NKG2D-mediated ADCC and direct killing (27).

Early in HIV-1 infection, directly preceding peak viremia, an extensive HIV-1 specific cytokine production is seen including factors known to directly influence NK cell effector function, phenotype, and/or proliferation (31, 32), such as type I interferons and IL-15 (33). Indeed, during the hyper-acute phase of infection, NK cells are highly active and cytolytic (10). In the same longitudinal single cell RNA sequencing study by Kazer et al. (10), two individuals that showed low viremia in chronic infection possessed cytotoxic and proliferating NK subsets (out of four persons tested). Pohlmeyer et al. (34) identified a subset of CD56dim CD16+ NK cells in EC that express CD11b, CD161, and Siglec-7 but not CD57. Based on marker expression and their increased effector function after *in vitro* stimulation with IL-12 and IL-18, the subset was defined as partially mature, highly active, and cytotoxic (34). This interestingly overlaps with findings of Kazer et al. (10) who additionally showed that NK cells from low viremia individuals produced the HIV-1 coreceptor CCR5 ligands CCL3 and CCL4 with anti-HIV-1 properties (35) during the early phases of infection. Adding to this are the observations that both chemokines are elevated in plasma of EC compared to viremic progressors - a fact previously attributed to CD8+ T cells (36–38). NK cells, however, are potent chemokine producers as well (39) and potentially contribute to viral control in EC through production of antiviral chemokines.

### Myeloid Cells Relay Immune Activation, but also Contribute to HIV-1 Persistence

Monocytes and macrophages are myeloid-derived innate immune cells forming the first barrier against pathogens by detecting them through pattern recognition receptors (PRR). Pathogen recognition triggers phagocytosis and initiates cytokine production relaying the danger signal to other immune cells (40, 41). Monocytes and macrophages then aid in tissue repair representing highly plastic functionality through reversibly changing their activation state (42). Both cell types are able to detect HIV-1 nucleic acids or proteins *via* different surface or intracellular Toll-like receptors (TLR) that mediate activation into a pro-inflammatory state (43).

Monocytes circulate the peripheral blood, whereas macrophages specialize and occupy tissues. Monocytes are subdivided into three groups. Classical monocytes (CD14+ CD16-) form the majority, whereas pro-inflammatory intermediate (CD14+ CD16+) and non-classical (CD14lo/- CD16+) monocytes represent a much smaller portion of all circulating monocytes (40). Most likely, they differentiate linearly in the given order (classical - intermediate - non-classical) with the last step potentially taking place outside of circulation before re-entering the bloodstream (44). When monocytes are activated by pathogens or inflammatory cytokines, they are able to migrate into tissue and acquire a macrophage-like phenotype (also referred to as infiltrating macrophages) supporting the tissue-resident macrophage population (41).

Macrophages are susceptible to productive HIV-1 infection and contribute to viral persistence (45). The permissiveness to infection, however, varies with the site and activation phenotype of the macrophage population (46) and is reduced in monocyte-derived macrophages of EC *in vitro* (47). HIV-1 infection of macrophages skews them towards a pro-inflammatory and dysfunctional phenotype (48, 49). Years of research indicate that HIV-1 infected and bystander macrophages contribute towards an inflammatory milieu and may be a driving force behind tissue damage (46, 50).

The establishment of an HIV-1 reservoir in tissue-resident macrophages plays an important part in viral persistence (51) and is thought to happen early in infection (52, 53). Despite the small size of the macrophage reservoir in HIV-1 positive individuals on ART viral reactivation in latently infected macrophages has the potential to cause rebound viremia (40, 52, 53). In CD4+ T cells of EC, however, it was demonstrated that reservoir cells have distinct proviral integration sites that silence viral genes (6, 54). However, characteristics of the macrophage reservoir in EC have not been uncovered to this date, and it remains to be answered whether the HIV-1 reservoir in macrophages is differently constituted in EC.

Monocyte-derived macrophages were shown to harbor HIV-1 in virus-containing compartments (VCC) supporting cell-to-cell spread of virions to T cells and thereby contributing to viral spread *in vitro* (55). The contribution of macrophage VCC to viral transmission *in vivo* is not known, but for EC it was shown that *SIGLEC1*, which is important for the formation of VCC (55), was downregulated in peripheral blood mononuclear cells hinting at a potentially impaired formation of VCC with better viral control (36, 46, 56).

HIV-1 infected macrophages are relatively resistant towards killing by cytotoxic lymphocytes like NK cells and CD8+ T cells. Their killing is dependent on granzyme B-mediated apoptosis *via* caspase 3, which can be affected by macrophage expression of the granzyme B inhibitor *SERPINB9* (57, 58). The inefficient killing of macrophages was further linked to the perpetuation of possibly damaging inflammatory processes (57, 58). Interestingly, CD4+ effector and CD8+ T cells from EC were able to inhibit viral replication in HIV-1 infected macrophages *in vitro*, though only CD8+ T cells were able to do so at a higher efficiency compared to chronic progressors on ART (59). The efficient delivery of lytic granules to HIV-1 infected CD4+ T cells by CD8+ T cells from EC is associated with HIV-1 control (60). A similar mechanism may also be involved in the increased CD8+ T cell mediated killing of HIV-1 infected macrophages seen in EC.

HIV-1 infection of circulating monocytes, on the other hand, has been a debated topic. There is evidence that monocytes are infected with HIV-1 *in vivo* based on the detection of intracellular HIV-1 DNA, however, the isolation of replication-competent viruses from monocytes has not been achieved and therefore open questions towards the nature of HIV-1 infection in monocytes remain (40). Apart from this, a central role for a proviral reservoir in monocytes of EC is unlikely because Spivak et al. (61) were not able to detect significant amounts of HIV-1 DNA in circulating monocytes of elite controllers.

In the acute phases of HIV-1 infection, monocytes are activated, expand, and show a prolonged upregulation of HLA class II molecules responsible for antigen presentation (10, 62). There is evidence for a polyfunctional monocyte phenotype with the expression of antiviral as well as inflammatory gene sets associated with low viremia in chronic infection (10).

Although the expansion of pro-inflammatory intermediate monocytes was reported for HIV-1 controllers and non-controllers alike, a higher frequency of non-classical monocytes was seen in the controller group (38, 63). When comparing EC to healthy individuals, however, one study did not see differences in the monocyte subset composition (38), whereas Krishnan et al. (64) detected a higher frequency of intermediate monocytes specifically in EC. They further found monocytes from EC to be more prone to *ex vivo* activation indicated by increased production of the pro-inflammatory cytokine IL-1β upon *in vitro* stimulation with the TLR4 ligand lipopolysaccharide (LPS) (64). Soluble CD14 and CD163, markers for monocyte activation, are associated with poor HIV-1 prognosis (65), can persist at high levels into chronic infection despite ART (62, 66, 67) and are also detected in EC (68–70). Although plasma viral load and monocyte activation as well as other inflammatory markers were not correlated in EC (68), increased inflammation may cause serious non-AIDS events, such as cardiovascular disease (70). However, in another study, elevated levels of sCD14 and sCD163 did not coincide with a higher prevalence for cardiovascular disease when comparing EC to HIV-1 negative individuals (69).

### Innate Immune Crosstalk

Crosstalk between the immunological first responders (monocytes in blood, macrophages in tissue, and NK cells) is important to orchestrate the innate immune response against pathogens (71, 72). Upon pathogen recognition, monocytes and macrophages release soluble factors and express ligands that activate NK cells and boost their effector function (72).

It was shown that pro-inflammatory macrophages stimulated with LPS prime NK cells *in vitro* for enhanced cytolytic function and cytokine production. Bellora et al. (73) reported increased NK activation and cytotoxicity after pro-inflammatory macrophage co-culture to be mainly dependent on soluble factors, whereas the production of IFNγ by NK cells was dependent on NK receptors 2B4 and DNAM-1 interacting with their ligands on macrophages in addition to IL-18 production by macrophages. The 2B4-CD48 axis was repeatedly implicated in enhanced IFNγ production of macrophage-primed NK cells (74, 75), and is additionally linked to NK cell proliferation (74).

Soluble factors secreted by pro-inflammatory macrophages that affect NK cell cytotoxicity were further characterized and involved (i) IL-23 and IFN-β to upregulate NKG2D, (ii) IL-1β to increase expression of NKp44, and (iii) the *trans*-presentation of IL-15 by macrophages (75). IFN-β also increased the *cis*-presentation of IL-15 by NK cells and in this way additionally triggered IFNγ production (75). In mice, it was demonstrated that monocytes, in an IL-15 dependent way, are important for NK cell differentiation into a more mature and cytotoxic phenotype (76). In humans, NK cells were shown to interact with TLR-stimulated monocytes *via* an activating NK receptor called NKp80 that binds CLEC2B, which reciprocally activated both cell types and increased the lytic function of NK cells (77). Additionally, the interaction of NK cells with NKG2D ligands on LPS-stimulated monocytes *via* NKG2D was implicated in IFNγ production of NK cells while having no effect on proliferation and cytotoxicity (78).

Virus infection of macrophages, as seen in HIV-1, may thus impact NK cells in two ways: as a trigger for direct NK cell killing of macrophages and as a possibly altered priming partner.

Lassa and Mopeia virus-infected macrophages, for example, were shown to activate NK cells *via* contact-dependent signals and type I interferons leading to an upregulation of NKp30 and NKp44 on NK cells, in addition to increasing NK cell proliferation and cytotoxic function (79). Nevertheless, NK cells primed by infected macrophages were not able to resolve macrophage infection but instead bidirectionally enhanced the activation of infected macrophages (79). When interacting with HIV-1 infected macrophages, NK cells are skewed towards producing the pro-inflammatory cytokine TNFα and seemingly switch their mode of killing macrophages from rapid granzyme-based cytotoxicity to slower death-receptor mediated apoptosis (57). To date, the contribution of effective NK cell priming by macrophages to elite control of HIV-1 infection is not understood.

## Discussion

A vast majority of HIV-1 positive individuals are dependent on ART to control HIV-1 progression and in many parts of the world the availability of treatment remains insufficient. The study of EC has helped to uncover factors important for natural control of HIV-1 infection (see **Table 1**). Nonetheless, EC are a heterogeneous group and likely achieve viral control in different ways (1). Recent reports highlighted that although natural control of HIV-1 replication is achievable for some, the possibility of long-term progression towards AIDS or chronic comorbidities in these individuals cannot be excluded (80). It is consequently of importance to fully unravel the mechanisms leading to long-term immune control of HIV-1.

**Table 1:** NK cell and monocyte/macrophage characteristics associated with HIV-1 control.

Cell type	Reported characteristics	Sample size	Reference no.
NK cells	High expressing KIR3DL1 variant and HLA-Bw4-I80 associated with increased degranulation against target cells and cytokine production after stimulation *in vitro*	n = 13 EC	(20)
	KIR3DL1 variant I47V enhanced protective effect of HLA-B*57:01	n = 297 LTNP*, n = 213 progressors	(4)
	Activated/cytolytic phenotype with expression of NKG2D and HLA-DR but no upregulation of NKp44 after IL-2 stimulation *in vitro* compared to progressors and healthy controls	n = 31 LTNP* (from this: n = 15 EC), n = 25 progressors, n = 10 HD	(24)
	Higher NKG2D expression compared to chronic progressors	n = 9 LTNP*, n = 56 progressors, n = 12 LTNP*, n = 12 progressors	(28, 29)
	CD56dim CD16+ subset with expression of CD11b, CD161, and Siglec-7 but not CD57 and increased effector function after *in vitro* stimulation with IL-12 and IL-18	n = 13 EC	(34)
	Elevated plasma CCL4 compared to progressors	n = 19 EC, n = 8 progressors	(36)
	Elevated plasma CCL3 and CCL4 compared to healthy donors	n = 3 EC, n = 9 HD	(37)
	Elevated plasma CCL4 compared to healthy donors	n = 14 EC, n = 12 HD	(38)
Macrophages	Reduced permissiveness to HIV-1 infection in monocyte-derived macrophages *in vitro*	n = 12 LTNP*, n = 11 HD	(47)
	More efficient inhibition of viral replication in monocyte-derived macrophages by CD8+ T cells of EC compared to progressors *in vitro*	n = 12 EC, n = 11 progressors	(59)
Monocytes	No HIV-1 DNA detectable in circulating monocytes	n = 11 EC	(61)
	Expansion of intermediate and non-classical subset in controllers	n = 20 LTNP*	(63)
	No differences in subset frequencies compared to healthy controls	n = 14 EC, n = 12 HD	(38)
	Higher frequency of intermediate subset in EC; Increased production of IL-1β upon *in vitro* stimulation with LPS	n = 26 EC, n = 18 HD	(64)
	Increased plasma sCD14 compared to progressors and healthy controls	n = 42 EC, n = 80 progressors, n = 43 HD	(68)
	Increased plasma sCD14 and sCD163 compared to healthy controls	n = 210 LTNP* (from this: n = 30 EC), n = 499 HD	(69)
	Increased plasma sCD163 compared to progressors and healthy controls	n = 10 EC, n = 103 progressors, n = 49 HD	(70)

EC, elite controllers; LTNP, long-term non-progressors; HD, healthy donors. *Definition of LTNP varies depending on study.

In concordance with CD8+ T cells, increased cytolytic and cytokine producing activity of NK cells next to certain receptor-ligand interactions may contribute to rapidly establish viral control in some HIV-1 positive individuals (12). The factors that influence enhanced NK cell activity in the context of HIV-1 control remain to be determined, but a defined cytokine milieu including IL-15 and a mature and cytotoxic NK cell subset secreting antiviral chemokines may be of importance. Protective HLA class I alleles in combination with certain NK cell receptors may render infected cells more sensitive to NK cell recognition. The involvement of cytotoxicity mediated by NKG2D (27–29) and the selective silencing of NKp44 (24) in controllers offers further clues towards viral control. It also indicates that the role of NK cells likely extends from the acute phase to long-term control in chronic HIV-1 infection.

There are different characteristics of monocytes and macrophages associated with low viremia or HIV-1 control. Among those are early monocyte activation, polyfunctionality, and, although conflictingly reported, specific monocyte subpopulations.

A reduced permissiveness to infection and a potentially altered formation of the VCC in macrophages of EC may represent ways in which macrophages directly affect viral load.

In a broader view, monocytes and macrophages are both integral cytokine producers in HIV-1 infection (9), which can impact adaptive responses as well as NK cell function (71). Currently, it is not known whether monocytes and macrophages of HIV-1 EC can prime NK cells more effectively compared to chronic progressors, or whether the crosstalk between these innate effectors predominantly drives inflammation. Interestingly, it was shown that NK cells of EC express high levels of NKG2D involved in killing of HIV-1 infected CD4+ T cells (27–29), which may be promoted by macrophage-derived IL-23 and IFN-β (75). NK cells from EC also have a higher sensitivity towards IL-18 in a highly active and cytotoxic subset (34) speculatively rendering them more susceptible to priming by pro-inflammatory macrophages (73).

NK cell development and function highly depend on IL-15 (31). The involvement of IL-15 dependent mechanisms in the priming of NK cells by monocytes/macrophages therefore has the potential to boost NK cell function against HIV-1 infected cells significantly (81). IL-15 is also one of the highest elevated cytokines just before peak HIV-1 viremia (33). There are currently two clinical trials ongoing (ClinicalTrials.gov Identifier: NCT04505501; NCT04340596) evaluating the impact of an IL-15 super-agonist in HIV-1 disease progression as a potential treatment to increase NK cell cytotoxicity, survival, and maturation (31) and thereby enhance viral control. However, later in infection, high IL-15 levels are linked to increasing viremia and inflammation (82), suggesting that resolution of the cytokine trigger is needed to prevent inflammation-associated pathology in HIV-1 and that, with respect to IL-15 dependent treatment options, careful assessment is required.

Elite controllers efficiently control HIV-1 – which potentially comes at a cost of increased inflammation driving non-AIDS-related comorbidities. Their immune system is possibly the closest model for functional HIV-1 cure, and therefore it is important to continue investigating the immunological mechanisms leading to this unique status. It remains to be further elucidated how the interaction between monocytes/macrophages and NK cells is orchestrated in HIV-1 infection and which aspects benefit short- and long-term viral control.

## Author Contributions

LM wrote the first draft of the manuscript, AH has made substantial, direct, and intellectual contributions to the work and all authors approved it for publication.

## Funding

AH was supported by the DZIF (German Center for Infection Research, TTU 01.709) and *via* the Clinician Scientist Program of the Faculty of Medicine, University Medical Center Hamburg-Eppendorf, Hamburg, Germany.

## Conflict of Interest

The authors declare that the research was conducted in the absence of any commercial or financial relationships that could be construed as a potential conflict of interest.

## Publisher’s Note

All claims expressed in this article are solely those of the authors and do not necessarily represent those of their affiliated organizations, or those of the publisher, the editors and the reviewers. Any product that may be evaluated in this article, or claim that may be made by its manufacturer, is not guaranteed or endorsed by the publisher.
